# OGG1 in the Kidney: Beyond Base Excision Repair

**DOI:** 10.1155/2022/5774641

**Published:** 2022-12-30

**Authors:** Fan Zhao, Jiefu Zhu, Lang Shi, Xiongfei Wu

**Affiliations:** ^1^Department of Nephrology, Renmin Hospital of Wuhan University, Wuhan, Hubei Province, China; ^2^Department of Organ Transplantation, Renmin Hospital of Wuhan University, Wuhan, Hubei Province, China

## Abstract

8-Oxoguanine DNA glycosylase (OGG1) is a repair protein for 8-oxoguanine (8-oxoG) in eukaryotic atopic DNA. Through the initial base excision repair (BER) pathway, 8-oxoG is recognized and excised, and subsequently, other proteins are recruited to complete the repair. OGG1 is primarily located in the cytoplasm and can enter the nucleus and mitochondria to repair damaged DNA or to exert epigenetic regulation of gene transcription. OGG1 is involved in a wide range of physiological processes, such as DNA repair, oxidative stress, inflammation, fibrosis, and autophagy. In recent years, studies have found that OGG1 plays an important role in the progression of kidney diseases through repairing DNA, inducing inflammation, regulating autophagy and other transcriptional regulation, and governing protein interactions and functions during disease and injury. In particular, the epigenetic effects of OGG1 in kidney disease have gradually attracted widespread attention. This study reviews the structure and biological functions of OGG1 and the regulatory mechanism of OGG1 in kidney disease. In addition, the possibility of OGG1 as a potential therapeutic target in kidney disease is discussed.

## 1. Introduction

Renal inflammatory diseases are a group of diseases that occur in the kidney and involve the inflammation of cells, including obstructions, tumors, metabolism, heredity, or injury [1]. Acute kidney injury (AKI) is the clinical manifestation of most acute kidney diseases, with chronic kidney disease (CKD) being the final outcome [2]. Although there has been a great deal of advancements in the understanding of biomarkers for the clinical diagnosis of AKI and advances in pathophysiological understanding, the transformation of molecular-based research into clinical therapy lacks effective targets. The multifactorial etiology of AKI and the complexity of the patient population introduces spatiotemporal and individual differences into the search for effective treatments [3]. In hospital settings [4], renal ischemia-reperfusion injury due to sepsis, surgery, trauma, or nephrotoxin injury is the leading cause of AKI. However, infections, toxins, and dehydration associated with acute illness are common causes of community-acquired AKI. Under oxidative stress conditions [5], reactive oxygen species (ROS) [6] or toxin [7] stimulation aggravates the damage to renal tubular epithelial cells (TECs) and vascular endothelial cells, further activating inflammatory pathways [8]. This leads to an imbalance of cellular homeostasis and abnormal repair [9], eventually leading to irreversible kidney damage and progression to CKD or renal tumors. Therefore, identification of novel effective targets and agents is of great importance for the prevention and treatment of kidney injury.

ROS, the most common cell damage factor in AKI, can be generated either externally or through the cellular environment from the metabolism of cells themselves [10]. Guanine has a low-redox potential [11] due to its unsaturated N7-C8 bond and is easily oxidized by reactive oxygen species to produce its most common oxidation product, 8-oxoG. 8-oxoG constitutes the most frequent base lesion observed in DNA, with an estimated frequency of 0.3–4 lesions per 106 bases [12]. The arrangement of the H-bond donor and acceptor is changed in 8-oxoG, and this characteristic miscoding gives 8-oxoG its special mutagenic properties [13]. In addition to Watson-Crick pairing with cytosine, 8-oxoG can form a stable Hoogsteen pairing with adenine, which can lead to a G:C to T:A conversion after replication [14]. Considering the high-mutagenic potential of 8-oxoG, 8-oxyguanine DNA glycosylase is produced in living organisms. The first step of the catalytic basic excision repair pathway is the detection and removal of 8-oxoG from oxidative DNA damage [15]. Removal of 8-oxoG through the action of OGG1 is essential to prevent genomic instability and allow correct gene transmission from one generation to the next.

OGG1 can bind to promoter regions rich in 8-O guanine, causing changes in DNA conformation, the recruitment of transcription factors, and the activation of downstream gene transcription [16]. The mechanism of OGG1 involvement in renal inflammatory diseases is as follows: OGG1 can enhance NF-*κ*B/RelA binding to cis-elements and thereby induce the rapid expression of chemokines/cytokines and inflammatory cell accumulation in airways [17]. OGG1 also directly interacts with other pathway proteins and affects downstream biological processes. OGG1 promotes TGF-*β*1-induced cell transformation through interacting with Smad7, activates SMAD2/3, and promotes fibrosis by interacting with the TGF-*β*/Smad axis [18]. Recent studies have indicated that OGG1 participates in autophagy and mitophagy regulation [19]. In hyperoxygen-induced lung injury, OGG1 interacts with molecular proteins of the autophagy pathway to negatively regulate the release of inflammatory cytokines [20].

OGG1 has been extensively studied in tumors, but less commonly in renal tumors. OGG1 is a common mutation site in renal tumors such as renal clear cell carcinoma [21]. Chronic kidney disease (CKD) is a multifactorial chronic disease characterized by genetic abnormalities. Studies have found that the OGG1 gene is associated with DNA damage in patients with CKD, and that OGG1 may be involved in the pathological process of CKD [22]. Studies have indicated that OGG1 promotes fibrosis progression in CKD by interacting with Smad7 to promote TGF-*β*1-induced cell transformation [18].

## 2. Structural Features and Biological Functions of OGG1

OGGs belong to the helix-hairpin-helix superfamily of base excision repair DNA glycosylases [23]. The helix-hairpin-helix structure [24] is comprised of amino acids at position 245-270 which serves as the catalytic region of the OGG family. OGGs also contain a glycine/proline-enriched region and a conserved aspartic acid motif. This motif contains lysine 249 and aspartic acid 268 and binds DNA to exert the catalytic activity of OGG1. OGG members are divided into three subfamilies: OGG1, OGG2, and AGOG. OGG1 exists mainly in eukaryotes and a few bacteria [25]. OGG2 is present in bacteria and archaea, while AGOG is present in archaeal organisms. The most significant difference between OGG1, OGG2, and AGOG is the additional N-terminal A domain, which is formed by an antiparallel twisted *β*-slice and is found only in the OGG1 enzyme. Since OGG2 and AGOG enzymes can cleave 8-oxoG from DNA in the absence of the A domain, this suggests that the N-terminal domain has other functions that are not specifically enzymatic digestive functions. In addition, human OGG1 is present as two major alternative splice isomers, hOGG1*α* and hOGG1*β* [26], which have different C-terminal domains. The human OGG1*α* isoform is expressed in the cytoplasm, nucleus, and mitochondria, whereas the human OGG1*β* isoform is only expressed in the mitochondria. The absence of mitochondrial translocation signals in the A domain of the hOGG1 N-terminus seems to prevent its localization to mitochondria [27] and supports the role of the N-terminal A domain in protein localization. The STRING database lists several protein-protein interactions of hOGG1, including protein kinase C, XRCC1, and PARP1 [28]. Therefore, the N-terminal A domain of OGG1 may be an anchor point involved in protein interactions.

OGG1 is a DNA glycosylase enzyme with apurinic/apyrimidinic (AP) site lytic activity which removes ROS-induced 8-oxoG [29]. OGG1 can bind to the promoter region of the inflammatory cytokine Cxcl2 [30] to activate transcription independently of its digestion activity. Various protein-protein interactions may modulate OGG1 activity. For example, in *in vitro* experiments, with an increase in APE1 [31], OGG1 AP lyase activity increased, and the binding and modification of OGG1 to PARP1 [32] reduced BER function. OGG1 can regulate gene transcription of the fibrosis factor VEGF through the presence of a putative G-quadruplex sequence in the promoter of the binding VEGF-coding chain [33]. OGG1 binds to oncosuppressor gene promoters and recruits chromodomain helicase-DNA-binding protein 4 (CHD4), which is associated with cancer. Finally, OGG1-mediated RAS activation can induce MEK, ERK, and PI3K to activate the NF-*κ*B signaling pathway and induce downstream inflammatory gene expression [34, 35].

## 3. OGG1 in Cellular Homeostasis

**Figure 1:**
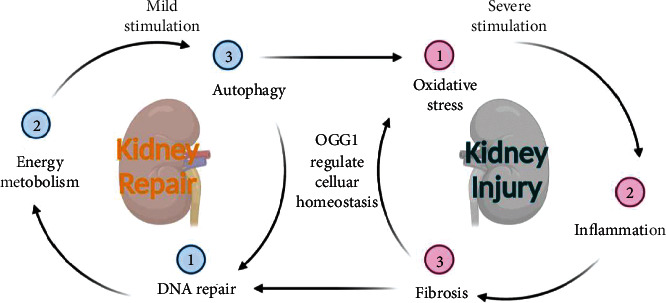
Biological functions of OGG1. OGG1 can regulate DNA repair, energy metabolism, and autophagy pathway in response to mild stimulation and regulate oxidative stress, inflammation, and fibrosis exposed to severe stimulation. OGG1: 8-oxyguanine DNA glycosylase 1.

OGG1 is mainly located in the cytoplasm and can enter the nucleus and mitochondria to repair DNA and activate multiple transcriptional pathways to regulate cellular homeostasis during cell injury. OGG1 regulates homeostasis through a variety of pathways, including DNA repair [36], oxidative stress [37], inflammatory responses, fibrosis, mitophagy [38], apoptosis, and energy metabolism (Figure 1). In addition to conventional DNA base excision and repair, OGG1 regulates downstream gene transcription as a key regulator of cellular homeostasis, mainly in the following three ways: (1) G-quadruplexes mediated by the BER pathway affect gene expression. (2) The BER pathway recruits topoisomerase to promote gene expression. (3) OGG1 recruits chromatin modification complexes to influence gene expression.

## 4. DNA Repair

**Figure 2:**
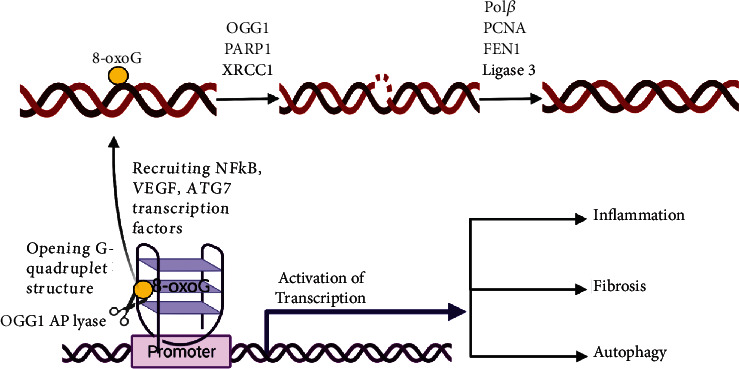
The role of OGG1 in regulation gene transcription in the promoter region. ROS oxidizes guanine in the DNA promoter region and recruits OGG1 to DNA promoter region, and OGG1 interacts with PARP1 and XRCC1 to remove 8-oxoG. At the same time, the G-quadruplex structure is opened up and the transcription factors NF-*κ*B, VEGF, and ATG7 are recruited to activate downstream inflammatory, fibrosis, and autophagy pathways. Then, Pol*β*, PCNA, FEN1, and Ligase 3 work together to repair DNA double strands. ROS: reactive oxygen species; PARP1: polyADP-ribose polymerase 1; XRCC1: X-ray repair cross complementing 1; NF-*κ*B: nuclear factor kappa-B; VEGF: vascular endothelial growth factor; ATG7: autophagy-related 7; Pol*β*: DNA polymerase*β*; PCNA: proliferating cell nuclear antigen; FEN1: flap structure-specific endonuclease 1.

The main types of DNA damage [39] include base deletions, mismatches, DNA crosslinking, and DNA strand breaks, which consist of DNA single-strand breaks (SSBs) and DNA double-strand breaks (DSBs). The base excision repair [40] pathway involves the repair of various types of DNA damage affecting the nuclear genome and is the most basic and important DNA repair method. OGG1 is a key enzyme that initiates the base excision repair pathway in prokaryotic and eukaryotic cells [36]. OGG1 has 8-oxoG DNA N-glycosylase activity [41]. OGG1 is also a double-edged sword in the process of base excision repair. Converting 8-oxoG into an AP site carries risk. First, there is the risk of information loss and mutations. Second, AP sites have strong effects on DNA secondary structure, protein binding, and G-quadruplex folding [42]. Third, AP sites stall transcription and replication [31] and the reactive aldehyde group of the AP site may react with amino groups to form DNA-protein crosslinks with potentially deleterious consequences for genome integrity [43]. Lastly, the related injured site and repair-associated conversion to single-strand breaks carry the risk of damage acceleration towards DNA double-strand breaks, resulting in genome instability, mutation, translocation, and loss of information. 8-oxoG accumulation may be connected to the DNA secondary structure of G-quadruplex folds, leading to higher sensitivity towards base modification or impaired excision by OGG1 in some secondary structures at the telomeres [44]. Antioxidant-mediated upregulation of OGG1 via NRF2 induction is associated with the inhibition of oxidative DNA damage [45] (Figure 2).

OGG1-deficient cells exhibit enhanced spontaneous mutagenesis [46, 47]. Upregulation of OGG1 may improve the ability of base excision repair to combat DNA damage and rescue genomic instability [48]. Upon PARP1 overexpression in cells, OGG1 forms an immunoprecipitable complex with PARP1, and inhibition of PARP1 or OGG1 results in DNA damage and decreased viability, which enhances DSB repair [49]. Under oxidative stress, OGG1 interacts with the mediator subunits CDK8 and MED12 on chromatin to maintain genomic stability [50]. Transcription-coupled nucleotide excision repair factor, Cockayne syndrome protein B (CSB), has been suggested to function in the repair of oxidative DNA damage. CSB promotes XRCC1 recruitment to oxidative DNA damage to maintain genome stability by OGG1 and interacts with PARP1 [51]. Chip assay results showed that when levels of 8-oxoG in the G-quadruplex structure of DNA were increased through H_2_O_2_ exposure, OGG1 was recruited to the KRAS promoter and further recruited the downstream nuclear factors MAZ and hnRNPA1, which are critical for transcription, reflecting the transcriptional regulation of OGG1 in the folding and stability of DNA promoter regions [52]. During gene damage, CHD4 helps maintain transcriptional silencing associated with DNA hypermethylation. CHD4 is recruited by OGG1 upon oxidative damage and interacts with 8-oxoG, which plays an important role in inhibiting tumor proliferation, invasion, metastasis, and in DNA stability [53]. Genome-wide mapping of AP site damage, BER protein binding, and G-quadruplex structures revealed that oxidative base-induced AP site damage was consistent with the binding and localization of OGG1 and APE1 in G-quadruplex structures, suggesting that the interaction between APE1 and OGG1 plays an important role in the regulation of G-quadruplex structure formation in the genome [54]. Additionally, acute oxidative stress leads to increased RECQL4 acetylation and its interaction with OGG1 participates in base excision repair. The NAD+-dependent protein SIRT1 deacetylates RECQL4 *in vitro* and in cells, thereby controlling the interaction between OGG1 and RECQL4 after DNA repair by maintaining RECQL4 in a low-acetylated state. The stimulation of human *α*-OGG1 catalytic activity by AP endonuclease-APE1 was proposed to enhance turnover and bypass of AP lyase activity [55]. Owing to the interaction between OGG1 and APE-1, stimulation of human *α*-OGG1 activity was also observed in the presence of the scaffold protein XRCC1 [56]. Human *α*-OGG1 also physically interacts with PARP1 to stimulate polyADP-ribosylation [32]. This suggests that OGG1 plays a critical role in DNA repair.

OGG1 knockout mice are viable, fertile, and do not show marked pathological defects in adulthood. However, a third study reported significantly higher lung tumorigenesis in OGG1 knockout mice at 18 months after birth than in WT mice. Furthermore, OGG1-/- mice accumulate 8-oxoG in the liver nucleus and mitochondrial DNA in an age-dependent manner [57]. Strong cancer susceptibility was observed in OGG1 and Mutyh double-knockdown mice, in which 8-oxoG accumulated in the liver, lung, and small intestine but not in the brain, kidney, and spleen, showing organ specificity [58].

Abnormal base excision repair pathways of OGG1 and OGG1 polymorphisms are associated with a variety of tumors in humans [59]. The OGG1 Ser326Cys polymorphism may be a risk factor for cancers of the lungs, digestive system, and head and neck [60]. Additionally, a significant association was observed between OGG1 germline mutations and breast cancer risk, which are considered promising targets for the diagnosis, treatment, and prevention of breast cancer [61]. Defective OGG1 regulates the coordination between innate and adaptive immunity through excessive oxidative stress and cytokine dysregulation, which are important targets in lung cancer treatment [62]. The OGG1 gene has somatic mutations in some human cancer cells, which are highly polymorphic in the human population. The repair activity of the mutant OGG1 protein is significantly lower than that of the wild-type form and is thus involved in many types of tumorigenesis [63].

## 5. Mitochondrial Homeostasis

The most important factor in maintaining the balance of mitochondrial homeostasis is the stable repair of mitochondrial DNA (mtDNA). OGG1, a key enzyme involved in mtDNA repair, is important for mitochondrial homeostasis. OGG1 decreases mitochondrial fragmentation and improves mitochondrial function in H9C2 cells under oxidative stress conditions [64]. The levels of the mitochondrial proteins involved in fission, DRP1 and FIS1, have been reported to be reduced in cells overexpressing mouse OGG1. Another study [65] found that overexpression of *α*-OGG1 protected the mitochondrial network from fragmentation after exposure to menadione. Targeting the human mutant protein MTS-OGG1-R229Q to the mitochondria results in decreased mtDNA integrity and cellular survival after exposure to oxidative agents when compared to the wild-type MTS-OGG1, and catalytically inactive *α*-OGG1 mutants did not preserve the mitochondrial morphology in the cells exposed to oxidative stress [66]. In addition, an increase in OGG1 acetylation, an increase in mitochondrial ROS, and a decrease in SIRT3 are all related to mtDNA deletion [67]. Mitochondria-targeted human 8-oxoguanine DNA glycosylase and aconitase-2 reduce oxidant-induced alveolar epithelial cell apoptosis, preventing oxidant-induced mitochondrial dysfunction, p53 mitochondrial translocation, and intrinsic apoptosis [68]. Nrf2 can bind to the antioxidant response element in the promoter of OGG1, participate in mtDNA repair, and maintain mitochondrial homeostasis [69].

## 6. Oxidative Stress

Under oxidative stress, the repair of 8-oxoG by the BER enzymes OGG1 and APE1 perturbs the structural equilibrium of the VEGF promoter DNA sequence between duplex and G-quadruplex structures, resulting in epigenetic modifications of gene expression [70]. Binding of APE1 to the AP site of OGG1 on the putative G-quadruplex sequence promoter element of VEGF enhances gene transcription to improve oxidative stress damage [71]. Studies have shown that OGG1 can activate the Nrf2 signaling pathway to protect renal tubular epithelial cells from oxidative DNA damage [72]. Long-term oxidative stress can lead to the continuous expression of OGG1-mediated inflammatory genes, leading to an excessive inflammatory response, which may lead to a series of diseases, such as cancer [73].

In addition to targeting DNA, oxidative stress can also affect proteins such as OGG1. Studies [74] have shown that under oxidative stress, OGG1 is sensitive to oxidants, and cysteine sites can be targeted to modify the response of OGG1 to alter its downstream cellular functions. The DNA repair function of OGG1 decreases under oxidative stress, and the main reason for this may be the cysteine-based enzymatic inactivation of OGG1. OGG1 can regain its repair activity after the redox balance is reestablished [75–77].

## 7. Inflammation

**Figure 3:**
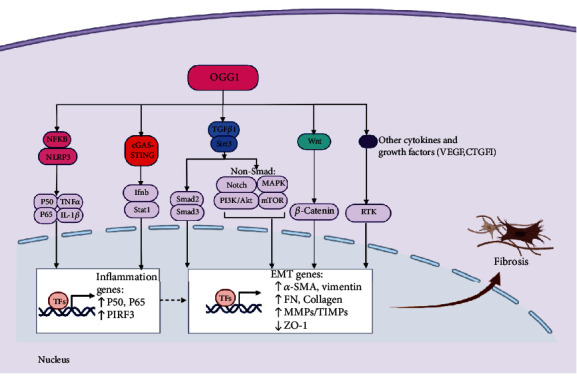
The role of OGG1 in the regulation of inflammation and fibrosis. OGG1 can act as a positive regulator of NF-*κ*B to prompt P50, P65, and TNF-*α* expression and further prompt the release of pro-IL-1*β* and the upstream inflammatory factors IL-1*β*. Furthermore, OGG1 regulates cGAS-STING pathway to increase the expression of the proinflammatory genes P50, P65, and PIRF3. Fibrosis is the main pathological process of various chronic diseases at the end stage and can be driven by inflammation. OGG1 can activate TGF-*β* to promote Smad2/3, notch, PI3K/AKT, and MAPK/mTOR signaling pathway and prompt the expression of fibrosis genes *α*-SMA, FN, and MMPs/TIMPs and downstream ZO-1. OGG1 can also positively regulate Wnt/*β*-catenin, VEGF, and CTGF, thus aggravating fibrosis. TNF-*α*: tumor necrosis factor-*α*; PIRF3: phosphointerferon regulatory factor 3; TGF-*β*: transforming growth factor beta; *α*-SMA: alpha-smooth muscle actin; FN: fibronectin; MMPs: matrix metalloproteinases; TIMPs: tissue inhibitors of metalloproteinases; ZO-1: zonula occludens-1. PI3K: phosphoinositide 3-kinase; MAPK: mitogen-activated protein kinase; mTOR: mammalian target of rapamycin; RTK: receptor tyrosine kinase.

OGG1 plays an important regulatory role in the immune inflammatory response. OGG1-deficient mice had good survival and showed significant resistance to acute and systemic inflammation. Visnes et al. [78] developed a selective active site inhibitor of OGG1, TH5487, which obstructs the binding and repair functions of OGG1 and 8-oxoG, and has obvious anti-inflammatory effects under the stimulation of oxidative stress, which is well-tolerated in mice. TH5487 prevents tumor necrosis factor-*α*-induced OGG1-DNA interactions at guanine-rich promoters of proinflammatory genes. NF-*κ*B is an important inflammatory transcription factor that enables downstream NLRP3 signaling and TNF-*α* expression, further promoting the release of IL-18, IL-1*β*, and other inflammatory cytokines [79]. This decreases the DNA occupancy of NF-*κ*B [80] and proinflammatory gene expression, resulting in decreased immune cell recruitment to the mouse lungs. Promoter-associated OGG1 enhances NF-*κ*B/RelA binding to cis-elements and facilitates the recruitment of specificity protein 1, transcription initiation factor II-D, and p-RNA polymerase II, resulting in the rapid expression of chemokines/cytokines and the accumulation of inflammatory cells in mouse airways [17]. OGG1-/- mice showed significantly higher expression of type I IFN genes such as Isg15, Irf9, and Ifnb. OGG1 regulates Ifnb expression through the cGAS-STING pathway [81]. Biochemical studies have shown that STAT1 plays a key role in endotoxin-induced OGG1 expression and inflammatory responses. OGG1 acts as a STAT1 coactivator and has transcriptional activity in the presence of endotoxins, resulting in the induction of the expression of proinflammatory mediators at the transcriptional level [82] (Figure 3).

## 8. Fibrosis

OGG1 appears to be involved in activating the WNT pathway and promoting the accumulation of nuclear *β*-catenin [83]. Studies [84] have shown that mtDNA damage and mutation are related to various pathological conditions, including the fibrosis of a variety of organs. Therefore, mtDNA repair is particularly important [85]. Deacetylase SIRT3 is located in the mitochondrial matrix and can bind to OGG1 in fibrosis, contribute to mtDNA repair, protect against apoptosis, and reduce fibrosis under oxidative stress [86]. In addition, SIRT3 can regulate OGG1 protein expression and activate DNA repair to prevent apoptosis and fibrosis [87, 88]. TGF-*β*1 is involved in the phenotypic transformation of fibroblasts. Induction of human lung fibroblasts with TGF-*β*1 increased the expression levels of fibrosis markers, smooth muscle *α*-actin collagen-1, and fibronectin [89]. TGF-*β*1 can also activate the PI3K/AKT [90] and MAPK [91] signaling pathways to regulate fibrosis. TGF-*β*1 treatment depletes SIRT3, further inducing increased production of ROS and DNA damage and decreased OGG1 levels [92]. However, overexpression of SIRT3 reverses the damage of fibrosis and induces mitophagy [93]. Inhibition of VEGF receptor signaling attenuates kidney microvascular rarefaction and fibrosis [94], and OGG1 can temporarily modify the hypoxia-response element of the VEGF gene under oxidative stress to regulate VEGF expression [95]. Although an association between OGG1 and the fibrosis pathway was found, the role of OGG1 in promoting fibrosis was more clearly demonstrated in the OGG1 knockdown mouse model. Wang et al. [18] observed that OGG1 promoted TGF-*β*1-induced cell transformation and activated Smad2/3 by interacting with Smad7 and that the interaction between OGG1 and the TGF-*β*/Smad axis modulates the cell transformation process in fibroblasts. Additionally, they demonstrated that OGG1 deficiency relieved pulmonary fibrosis and decreased the expression level of Smad7 and the phosphorylation of SMAD2/3 in BLM-treated mice (Figure 3). The role of OGG1 in promoting fibrosis is consistent with its role in promoting inflammation under oxidative stress as found by Visnes et al. [78], which further demonstrates the importance of OGG1 as a clinical target.

## 9. Autophagy

Autophagy is an important mechanism for cell homeostasis and is closely associated with cell repair. However, the exact relationship between DNA repair and autophagy remains unclear. OGG1 influences autophagy by binding to proteins or genes involved in the autophagy pathway. Ye et al. [20] found that OGG1 deficiency downregulates autophagy both *in vitro* and *in vivo* by decreasing lc3-I to LC3-II conversion, LC3 spot staining, and Atg7 expression. Additionally, they found that OGG1 binds to the promoter of Atg7 and that OGG1 can decrease the gene expression level of Atg7. Finally, OGG1 negatively regulates the release of inflammatory cytokines through molecular interactions that coordinate autophagy pathways in hyperoxygen-induced lung injury [96].

OGG1 can influence autophagy through several molecules that act as bridges. OGG1, p53, and TNF-*α* may jointly or independently repair DNA oxidative damage and/or induce apoptosis [97]. Studies have shown that DNA double-strand breaks can induce the coactivation of P53 and OGG1, and that they functionally coordinate [98]. The P53 protein is a key molecule in DNA damage-induced apoptosis and can play a bidirectional regulatory role in autophagy through its subcellular localization [99]. For example, P53 induces autophagy by activating AMPK, inactivating mTOR, and promoting the transcriptional expression of damage-regulated autophagy modulators [100–102]. Under glucose starvation, AMPK promotes autophagy by directly activating Ulk1 through the phosphorylation of Ser317 and Ser777. In response to injury, K63 ubiquitination of TAK1 activates AMPK in damaged lysosomes to trigger autophagy [103]. Under nutrient sufficiency, high-mTOR activity prevents Ulk1 activation by phosphorylating Ulk1 Ser757 and disrupting the interaction between Ulk1 and AMPK [104–106]. On the other hand, P53 can also promote the expression of phosphatidylinositol phosphate PTEN and then inhibit the PI3K/AKT/mTOR signaling pathway to activate autophagy [107]. Furthermore, Muñoz-Gámez et al. found that OGG1 binds directly to PARP1 through the N-terminal region of OGG1, and that this interaction is enhanced by oxidative stress [108]. PARP1 and OGG1 act in the same regulatory pathway and PARP1 activity is required for OGG1-mediated repair of oxidative DNA damage in G1-arrested cells. ROS-induced DNA damage and PARP1 are required for the optimal induction of starvation-induced autophagy [109]. Finally, PARP1 can induce ATP depletion and suppress the mTOR pathway to regulate autophagy initiation [110].

**Figure 4:**
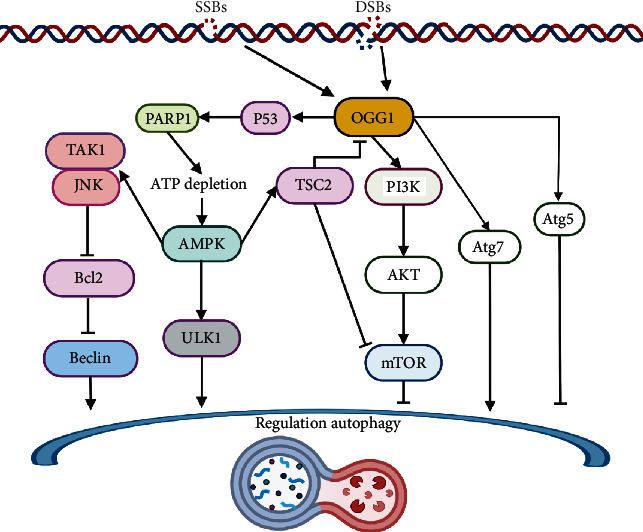
The role of OGG1 in the regulation of autophagy. DNA single- or double-strand oxidative damage recruits OGG1 excision repair 8-oxoG. OGG1 interacts with PARP1 and induces ATP depletion and the activation of AMPK. AMPK promotes TAK1/JNK pathway and ULK1 to regulate autophagy process. Additionally, OGG1 binds to the promoter region of ATG7 to induce autophagy. AMPK and TSC2 activation may negatively affect the expression of OGG1. On the other hand, OGG1 is found to inhibit autophagy by activating the PI3K/AKT/mTOR pathway in specific damage models. ATP: adenosine triphosphate; AMPK: adenosine monophosphate-activated protein kinase; TAK1: transforming growth factor *β*-activated kinase 1; JNK: c-jun N-terminal kinase; ULK1: Unc-51-like autophagy-activating kinase 1; TSC2: tuberous sclerosis complex 2.

Autophagy also regulates OGG1 expression and DNA repair. Some studies [111] have shown that autophagy can also promote the degradation of DNA damage repair proteins, thereby facilitating cell death. Other studies [112, 113] have shown that the inhibition of the autophagy pathway protein mTOR by rapamycin suppresses the repair of DSBs. In response to a nutrient starvation model, autophagy activation was shown to degrade OGG1 in cardiomyocytes [114]. This study found that OGG1 was activated by the autophagy inhibitor bafilomycin in autophagy-deficient Atg5(-/-) mouse cell models under nutrient deprivation. Nevertheless, the pharmacological activation of autophagy did not induce OGG1 loss. There may be a feedback mechanism between autophagy and OGG1 activation in specific injury models. In summary, the study of the relationship between OGG1 and autophagy is of great significance for exploring the regulatory relationship between DNA damage repair and autophagy, and many more studies are needed to fully explore this area (Figure 4).

## 10. Apoptosis and Energy Metabolism

The effect of OGG1 on apoptosis remains controversial. There is a view that mitochondria-targeted OGG1 overexpression can prevent mitochondria-regulated apoptosis caused by oxidative stress, including alveolar epithelial cell apoptosis following asbestos exposure [115]. However, after excessive oxidative damage, the BER pathway further increases the level of ROS-induced DNA damage by producing repair intermediates, leading to PARP1 overactivation and cell death [116]. OGG1-deficient mice exhibit excellent inflammatory resistance and a good survival ability, which seems to blur this controversy.

Researchers have found some metabolic differences in OGG1-deficient mice. Dr. Lloyd's laboratory found that OGG1+/+ OGG1-/- mice fed with a high-fat diet showed inconsistent gene expression in liver and muscle cells, suggesting that OGG1 may be related to gene expression in cellular metabolism [117]. After a high-fat diet for 12-week stress tests in relatively young animals, OGG1-/- mice became significantly heavier and accumulated more adipose tissue than WT counterparts. Levels of dynamin-related protein-1 and fission-1 were significantly increased in the muscles of OGG1-/- mice. In OGG1-overexpressing mice, following 12 weeks of a hypercaloric high-fat diet consumption, body weight and composition analyses revealed that OGG1 Tg mice were significantly protected from increased body weight and fat mass after the imposed high-fat diet relative to WT animals [118].

Chronic exposure to elevated levels of free fatty acids impairs pancreatic beta cell function and contributes to a decline in insulin secretion in type 2 diabetes. Overexpression of hOGG1 in the mitochondria can reduce FFA-induced inhibition of ATP production and reduce apoptosis of islet *β* cells, suggesting that OGG1 may be a new target for intervention in type 2 diabetes mellitus [119].

## 11. OGG1 in Kidney Disease

### 11.1. Kidney Tumors

The *OGG1* gene is located at 3p25 on chromosome 3, and a heterozygous deletion in the 3p25 region was found in 85% of 99 cases of renal tumor loci, including renal clear cell carcinoma. Hence, this is a common mutation site [21]. Chevillard et al. [120] identified homozygous mutations in renal tumors when screening for changes in *OGG1* cDNA in human tumors, including two transitions (GC to TA and TA to AT) and one transition (GC to AT) with a base substitution. All three of these substitutions led to amino acid changes in the human OGG1 protein.

In addition, the incidence of multiorgan tumors observed at 34 weeks in OGG1 knockout mice showed that the incidence of colon adenomas and total colon tumors showed a trend with a sharp increase, with bladder hyperplasia and an increased rate of tubular hyperplasia also observed [121]. Rapamycin-mediated activation of AMPK and inhibition of mTOR upregulates OGG1, which may be a viable therapeutic target for renal tumors [19]. Chromatin immunoprecipitation identified transcription factor AP4 as a positive regulator of the *OGG1* promoter. In the kidneys of patients with tuberous sclerosis, loss of a protein encoded by tuberous sclerosis complex 2 (Tsc2) can downregulate OGG1 protein expression by regulating transcription factor AP4 binding to the *OGG1* promoter, thereby increasing the incidence of tumors.

## 12. OGG1 in AKI

### 12.1. Ischemia-Reperfusion Injury

OGG1 has been widely studied in brain and lung ischemia-reperfusion injury but less so in renal ischemia-reperfusion injury. In a renal ischemia-reperfusion experiment in rats [122], high-performance liquid chromatography with mass spectrum analysis of the nuclear DNA revealed an immediate accumulation of 8-oxo-dG in nuclear DNA prepared from the cortex and outer medulla of the kidney 1 h after ischemia-reperfusion. An RNase protection assay showed a high level of OGG1 mRNA in the normal kidney, which decreased within 3 h only in the outer medulla and increased after 1-7 days of ischemia-reperfusion both in the cortex and outer medulla. They also found that the accumulation of 8-oxoG in mtDNA, rather than in nuclear DNA, may be involved in renal tubular cell injury and other pathological reactions caused by renal ischemia-reperfusion injury. In cardiac ischemia-reperfusion injury model studies, mtDNA damage repaired by OGG1 base excision seemed to have no significant effect on cardiac function [123, 124]. In an ischemia-reperfusion model, the DNA repair function of OGG1 is inhibited under oxidative stress, and the upregulated OGG1 levels may also have other transcriptional regulatory effects, which require further study.

The DNA repair function of OGG1 is decreased in ischemia-reperfusion injury, yet the function of inducing the NF-*κ*B pathway to activate the inflammatory response is not weakened. Inhibition of OGG1 with TH5487 interferes with OGG1 incision activity, resulting in fewer DNA double-strand breaks in cells exposed to oxidative stress and reducing the DNA mutation rate caused by OGG1 exposure to AP sites, which may reduce the incidence of cancer in cases of oxidative stress damage [125]. In addition, the anti-inflammatory function of TH5487 reduces inflammatory damage caused by oxidative stress and may contribute to ischemia-reperfusion in a more meaningful way [126].

### 12.2. Septic AKI

*Staphylococcus aureus* is an opportunistic pathogen and is one of the leading causes of life-threatening sepsis [127]. *Staphylococcus aureus* sepsis induces early renal mtDNA damage, upregulates the expression of inflammatory factors, such as TNF-*α*, IL-10, and Ngal mRNA, and activates OGG1 [128]. OGG1, an early septic mitochondrial reactive protein, is regulated by the nuclear transcription factors NRF-1 and NRF-2*α* and the activation of inflammation [129, 130]. Mabley et al. investigated the role of OGG1 in inflammation using an endotoxic shock model and found that OGG1-/- mice are resistant to endotoxin-induced organ dysfunction, neutrophil infiltration, and oxidative stress when compared with the response observed in wild-type controls (OGG1+/+) [131]. Furthermore, deficiency of OGG1 protects against inflammation and the mutagenic effects of *H. pylori* infection in mouse models [132]. These results indicate that OGG1 regulates the moderate activation of inflammation in septic AKI.

## 13. OGG1 in CKD

**Figure 5:**
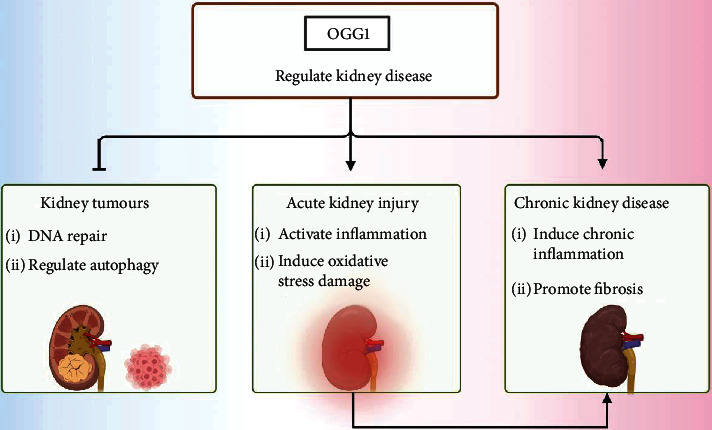
The role of OGG1 in kidney diseases. In kidney tumors, OGG1 induces DNA base excision repair and regulates autophagy to inhibit formation of kidney tumors. In acute kidney injury, OGG1 activates inflammation and induces oxidative stress to aggravate renal injury. In chronic kidney disease, OGG1 induces chronic inflammation and promotes fibrosis.

Chronic kidney disease (CKD) is a multifactorial chronic disease characterized by genetic abnormalities. Genome-wide association studies [133] have identified hundreds of loci in which genetic variants are associated with kidney function. However, causal genes and pathways involved in CKD remain unknown. In a study on the relationship between DNA damage, genomic instability, and gene polymorphisms in patients with CKD, it was found that genes such as *OGG1* and *XRCC1* involved in base excision repair are related to DNA damage [22]. OGG1 promotes fibrosis progression in chronic kidney disease by interacting with Smad7 to promote TGF-*β*1-induced cell transformation [18]. Another study showed the role of the Ser326Cys polymorphism in the *OGG1* gene, which modulates the level of 8-oxoG in the leukocytes of CKD patients [134]. OGG1 has also been widely studied in the context of tumors and lung injuries. Recent studies have found that the regulation of gene transcription of *OGG1* seems to be as important as the effect of base excision and repair [135]. How OGG1 regulates downstream gene transcription in patients with CKD is worthy of further exploration (Figure 5).

## 14. Conclusions and Prospects

Previous studies have shown that OGG1 inhibits tumorigenesis via base excision and repair. In recent years, studies have found that OGG1 participates in the regulation of renal inflammatory diseases by upregulating certain inflammatory cytokines, inducing oxidative stress and inflammation, promoting fibrosis, and regulating autophagy. The powerful anti-inflammatory effect of the OGG1 specific inhibitor TH5487 has been verified. As an emerging target, OGG1 has new prospects for the clinical treatment of inflammatory diseases of the kidney.

## Data Availability

The data will be available on request from the authors.
